# Unusual Large-Scale Chromosomal Rearrangements in *Mycobacterium tuberculosis* Beijing B0/W148 Cluster Isolates

**DOI:** 10.1371/journal.pone.0084971

**Published:** 2014-01-08

**Authors:** Egor A. Shitikov, Julia A. Bespyatykh, Dmitry S. Ischenko, Dmitry G. Alexeev, Irina Y. Karpova, Elena S. Kostryukova, Yulia D. Isaeva, Elena Y. Nosova, Igor V. Mokrousov, Anna A. Vyazovaya, Olga V. Narvskaya, Boris I. Vishnevsky, Tatiana F. Otten, Valery Y. Zhuravlev, Peter K. Yablonsky, Elena N. Ilina, Vadim M. Govorun

**Affiliations:** 1 Research Institute of Physical - Chemical Medicine, Moscow, Russian Federation; 2 Moscow Institute of Physics and Technology, Dolgoprudny, Russian Federation; 3 Moscow Scientific-Practical Center of Treatment of Tuberculosis of Moscow Healthcare, Moscow, Russian Federation; 4 St. Petersburg Pasteur Institute, St. Petersburg, Russian Federation; 5 Research Institute of Phthisiopulmonology, St. Petersburg, Russian Federation; Institut Pasteur de Lille, France

## Abstract

The *Mycobacterium tuberculosis* (MTB) Beijing family isolates are geographically widespread, and there are examples of Beijing isolates that are hypervirulent and associated with drug resistance. One-fourth of Beijing genotype isolates found in Russia belong to the B0/W148 group. The aim of the present study was to investigate features of these endemic strains on a genomic level. Four Russian clinical isolates of this group were sequenced, and the data obtained was compared with published sequences of various MTB strain genomes, including genome of strain W-148 of the same B0/W148 group. The comparison of the W-148 and H37Rv genomes revealed two independent inversions of large segments of the chromosome. The same inversions were found in one of the studied strains after deep sequencing using both the fragment and mate-paired libraries. Additionally, inversions were confirmed by RFLP hybridization analysis. The discovered rearrangements were verified by PCR in all four newly sequenced strains in the study and in four additional strains of the same Beijing B0/W148 group. The other 32 MTB strains from different phylogenetic lineages were tested and revealed no inversions. We suggest that the initial largest inversion changed the orientation of the three megabase (Mb) segment of the chromosome, and the second one occurred in the previously inverted region and partly restored the orientation of the 2.1 Mb inner segment of the region. This is another remarkable example of genomic rearrangements in the MTB in addition to the recently published of large-scale duplications. The described cases suggest that large-scale genomic rearrangements in the currently circulating MTB isolates may occur more frequently than previously considered, and we hope that further studies will help to determine the exact mechanism of such events.

## Introduction

The Beijing genotype of *Mycobacterium tuberculosis* (MTB) has been shown to be globally spread all over the world [1]. In Russia, half of the local MTB population belongs to Beijing genotype, and one-fourth of these strains belong to the so-called B0/W148 clonal group [2]. Members of this group possess a specific 17-band IS*6110* restriction fragment length polymorphism (RFLP) pattern, which was originally identified in Russia in the 1990s [3], [4]. In comparison with other Beijing genotypes, B0/W148 strains demonstrated an increased virulence in the macrophage model [5], a stronger association with multidrug resistance [6], and an increased transmissibility [7], [8]. The Beijing B0/W148 has been defined as a ‘successful Russian clone’ of *M. tuberculosis*, and its pathobiology and phylogeography have recently been reviewed and discussed [2]. Together, these findings have led to assumption that Beijing B0/W148 strains possess unique genomic features that gave them evolutionary advantages.

To date, a small amount of whole genome sequencing data for B0/W148 MTB strains has been uploaded into the international databases, including one genomic scaffold of the W-148 strain (GL877853.1) and raw sequencing data in the NCBI Sequence Read Archive for several strains from the Samara region in Russia [9]. The aim of this work was to get more profound knowledge regarding the properties of Beijing B0/W148 strains using comparative genomics approach. All newly sequenced genomes were shown to be similar to the W-148 strain. Whole genome alignment between W-148 and the reference H37Rv MTB strain revealed two large chromosomal inversions in the W-148 genome. The largest inversion changed the orientation of the three megabase (Mb) segment of the chromosome. The second one occurred in the previously inverted region and partly restored the orientation of the large inner segment. These two inversions were flanked by partial or complete copies of mobile genetic element IS*6110* and touched large parts of genome. Detailed PCR analysis of our sequenced strains (n = 4) revealed the rearrangements in their genomes identical to those ones found in W-148 strain.

Remarkably, only two cases of large-scale genome rearrangement events in the MTB have been reported until now. First case was reported by Domenech P. *et al*. [10] describing the duplication of a 350 kilobase (Kb) region spanning Rv3128c to Rv3427c in the strains belonging to the W/Beijing family of MTB lineage 2. The second case was described by Weiner B. *et al.* [11], showing the independent duplication events occurred in the MTB lineages 2 and 4 were found. We have found another example of chromosomal rearrangement, i.e. inversions of large DNA segments. Large inversions were previously detected in some bacteria [12], [13], but not in MTB.

Here we report two large-scale genome inversions characteristic exclusively for the members of MTB Beijing B0/W148 cluster and further hypothesize that these events occurred in their progenitor. This is the first report of a large-scale inversion in the MTB genome, and we hope that it will be one more step in filling the gap in the knowledge of a history and of an evolution of this pathogen.

## Results

### Genome sequencing of four clinical *M. tuberculosis* isolates belonged to Beijing B0/W148 cluster

**Figure 1:**
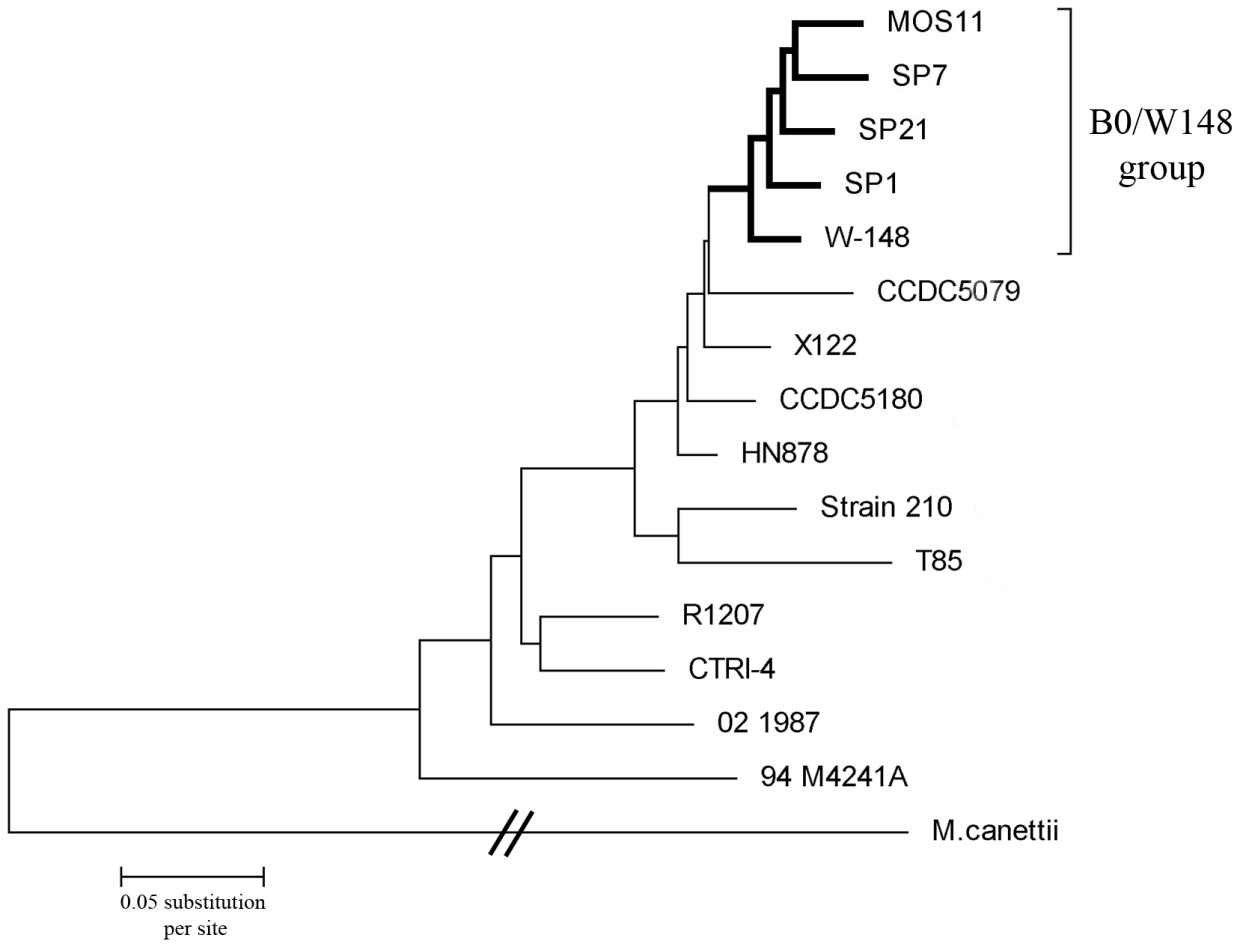
Comparative phylogenetic analysis of strains under study and 12 whole genomes from the NCBI database. Phylogenetic tree based on all SNPs of genomes was constructed using the Neighbor-Joining algorithm. Evolutionary distances were calculated using p-distance method.

Four Russian MTB isolates SP1, SP7, SP21, and MOS11 of the Beijing B0/W148 cluster were selected for whole genome re-sequencing (Table 1). Genomes were sequenced up to 98% completion using 454 pyrosequencing with more than 10-fold of coverage. To determine the taxonomy relationship between our strains and previously sequenced Beijing MTB strains deposited in GenBank, we have performed a phylogenetic analysis using polymorphisms relative to the reference genome of H37Rv MTB strain. The CTRI-4 strain previously sequenced in our laboratory and representing ancestral Beijing sublineage [17] was additionally included into analysis. Phylogenetic tree was built based on overall SNPs extracted from genomic DNA sequences after excluding SNPs for PE-PPE and PGRS protein families. This approach does not give us the perfect phylogenetic relationships in a case of fast evolving microorganisms influenced by recombination; however, it can be very efficient for the genetically monomorphic bacteria such as MTB [18]. The resulting phylogeny is shown in Figure 1. Phylogenetic tree demonstrated a close similarity between the genomes of four Beijing B0/W148 strains sequenced in this study and W-148 strain.

**Table 1:** Genotyping and drug resistance data of the B0/W148 strains sequenced in this study.

Strain	Drug resistance^1^	IS*6110* RFLP^2^	Spoligotype clade^3^ (SIT)	24-VNTR^4^	Location, year
SP1	RIF^R^, INH^R^, EMB^R^, STR^R^, PZA^S^, AMI^S^, CAP^S^, ETH^R^, OFL^R^	B0/W148	Beijing (1)	223325173533424672454433	Saint Petersburg (North West Russia), 2011
SP7	RIF^R^, INH^R^, EMB^R^, STR^R^, PZA^R^, ETH^R^, AMI^R^, CAP^R^, OFL^R^	B0/W148	Beijing (1)	223325173533424672454433	Saint Petersburg (North West Russia), 2011
SP21	RIF^R^, INH^R^, EMB^R^, STR^R^, PZA^S^, AMI^R^, CAP^R^, ETH^S^, OFL^R^	B0/W148	Beijing (1)	223325173533424672454433	Saint Petersburg (North West Russia), 2010
MOS11	RIF^R^, INH^R^, EMB^R^, STR^R^, PZA^S^, AMI^S^, CAP^S^, ETH^R^, OFL^R^	B0/W148	Beijing (1)	223325173533424672454433	Moscow (Central Russia), 2011

^1^ RIF - rifampicin, INH - isoniazid, EMB - ethambutol, STR - streptomycin, PZA - pyrazinamide, ETH - ethionamide, AMI- amikacin, CAPR - capreomycin, OFL – ofloxacin.

^2^ B0 designation according to Narvskaya *et al.* [6], W148 according to Bifani *et al.* [14].

^3^ SITVITWEB was used for identification of data [15].

^4^ 24 – VNTR: s154, s580, s960, s1644, s2059, s2531, s2687, s2996, s3007, s3192, s4348, s802, s2165, s2461, s577, s2163, s4052, s4156, s424, s1955, s2347, s2401, s3171, s3690 [16].

### Rearrangements in W-148 chromosomal DNA

**Figure 2:**
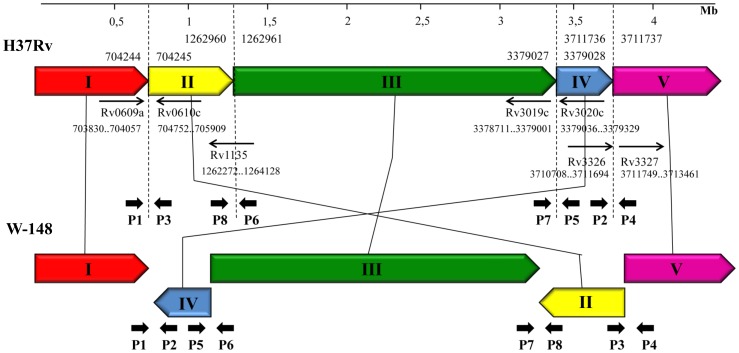
Schematic representation of genome rearrangements' and primer design strategy. Each local collinear block (LCB) corresponds to Table 2 and is represented by a different color.

Similarity of the genome sequences of the studied strains and W-148 gave us an opportunity to analyze structural genomic rearrangements within this group. Start of the W-148 genome was changed in relation to base 1 of the MTB H37Rv genomic sequence. This whole genome alignment of W-148 and H37Rv chromosomal DNA sequences revealed the presence of two large inversions in the W-148 genome. The Mauve 2.3.1 program highlighted these chromosomal rearrangements by subdividing the W-148 genome into five local collinear blocks (LCBs) (Table 2). This analysis demonstrated that the first, third and fifth LCBs were conserved whereas the second and forth were inverted and rearranged in W-148 with respect to H37Rv (Figure 2).

**Table 2:** Description of five local collinear blocks (LCBs) between H37Rv and W-148.

LCB	Type	H37Rv position	W-148 position
I	Conserved	1-704243	1-769418
II	Rearranged	704246-1262963	3247025-3814187
III	Conserved*	1262961-3379027	1108934-3245669
IV	Rearranged	3379025-3710381	773801-1107578
V	Conserved	3711737-4411532	3814961-4539434

- LCB III conserved according to initial mechanistic analysis, but re-arranged according to our hypothesis

### Chromosomal rearrangements in SP21 MTB strain confirmed by NGS

Based on similarity of genomic DNA sequences between SP1, SP7, SP21, MOS11 and W-148 strains, we expected to find the discovered inversions in the other strains as well. To confirm this, we additionally sequenced the SP21 strain using the mate-pair library strategy. The assembly of the SP21 genome sequence was performed by combining 454 (70 K reads, mean length 540 bp) and Ion Torrent data (650 K reads, 180 bp, mate-pair) that together represented more than 40-fold coverage of the genome. Initial assembly was performed by using the GS de novo Assembler and produced 391 contigs which length ranged from 500 to 69,788 bp. Further scaffolding resulted in 12 scaffolds with a total length of 4.45 Mb (AOUF00000000.1). The alignment of H37Rv, W-148, and SP21 chromosomal DNA sequences revealed the presence of identical large-scale inversions in both SP21 and W-148 stains (Figure S1).

### Chromosomal rearrangements in SP21 MTB strain confirmed by RFLP

The inversions observed in SP21 genome relative to H37Rv were verified by RFLP analysis. Based on analysis of the H37Rv restriction endonuclease map, the *Mlu*I was chosen for DNA digestion because its recognition sites were close to recombination junctions. The DNA probes specific to genome regions flanking the recombination junctions were obtained by PCR with specific primers (Table 1 in supplementary Text S1).

**Figure 3:**
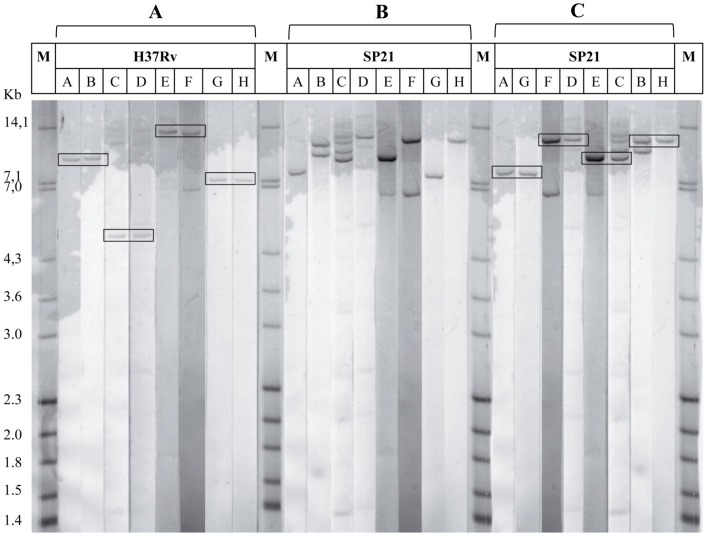
Southern blot analysis of H37Rv and SP21 MTB strains. Genomic DNA was digested with *Mlu*I and hybridized with the fluorescent labeled probes obtained by amplification. The probes are listed at the top of the lanes (from A to H). (A) Hybridization patterns of H37Rv strain. The order of probes corresponds to the order of complementary sequence sites in the genome of H37Rv. (B) Hybridization pattern of SP21 strains. The order of probes corresponds to the order of complementary sequence sites in the genome of H37Rv. (C) Hybridization patterns of SP21 strain. The order of probes is rearranged in accordance with the expected order of complementary sequence sites in the inverted genome (Supplementary Text S1). The merged bands from probes complementary to the boundaries of recombination junctions are boxed. M, marker strain Mt14323 (Mycobacterial Reference Laboratory, National Public Health Institute (Turku, Finland)).

The RFLP analysis was performed for both SP21 and H37Rv *M. tuberculosis* strains and revealed the rearrangements in the SP21 genome sequence relative to that of H37Rv (Figure 3). In case of H37Rv, the hybridization signals from A&B, C&D, E&F, and G&H probes perfectly matched each other (Figure 3A) and the size of RFLP fragments corresponded to the expected one (Figure 1 in supplementary Text S1). In case of SP21, the RFLP pattern was different (Figure 3B). The signals from alternative combinations of probes (A&G, F&D, E&C and B&H) matched each other, which indicated the presence of this specific inversion (Figure 3C). The RFLP fragments corresponded to those expected in the inverted genome.

### The presence of inversions in other members of Beijing B0/W148 and non-Beijing B0/W148 groups

To verify the presence of the discovered inversions in another Beijing B0/W148 MTB strains, we developed a set of PCR primers flanking the sites of inversions. All primers were designed on the basis of W-148 genome (Table 3). Two pairs (P1&P2, P3&P4) flanked the ends of the external inverted region (between LCB I&IV and LCB II&V, respectively); and other pairs (P5&P6, P7&P8) flanked the ends of the internal region (between LCB IV&III and LCB III&II, respectively) (Figure 2). The primers were designed in such a way that the same primers used in different combinations would be suitable for analysis of genomic arrangement in other, non-B0/W148 strains. The size of expected PCR products is shown in Table 3.

**Table 3:** Primers designed for analysis of genomic arrangement in studied MBT strains.

Primers' set No	Primer's name	5′-3′ sequences	Product's length		
			B0/W148 Beijing	other Beijing	non-Beijing (Ural, LAM)
	External inversion (links between LCB I, II, IV, V)				
1	P1	gtgttgtacattgggcatcg	1021	no reaction	no reaction
	P2	ggtgtacatatcgaagctcg			
2	P3	gctcgacgaagtgagattgc	2215	no reaction	no reaction
	P4	ttggcgatccgatacagtgc			
3	P1	gtgttgtacattgggcatcg	no reaction	320	320
	P3	gctcgacgaagtgagattgc			
4	P2	ggtgtacatatcgaagctcg	no reaction	2920	1845
	P4	ttggcgatccgatacagtgc			
	Internal inversion (links between LCB II, III, IV)				
5	P5	ctgccaagcactggacagc	1761	no reaction	no reaction
	P6	caagtctccggtattcaagg			
6	P7	agccttggctcgtccttacc	2527	no reaction	no reaction
	P8	cacggctctcccaacgtgg			
7	P6	caagtctccggtattcaagg	no reaction	1841	483
	P8	cacggctctcccaacgtgg			
8	P5	ctgccaagcactggacagc	no reaction	2447	1090
	P7	agccttggctcgtccttacc			

**Figure 4:**
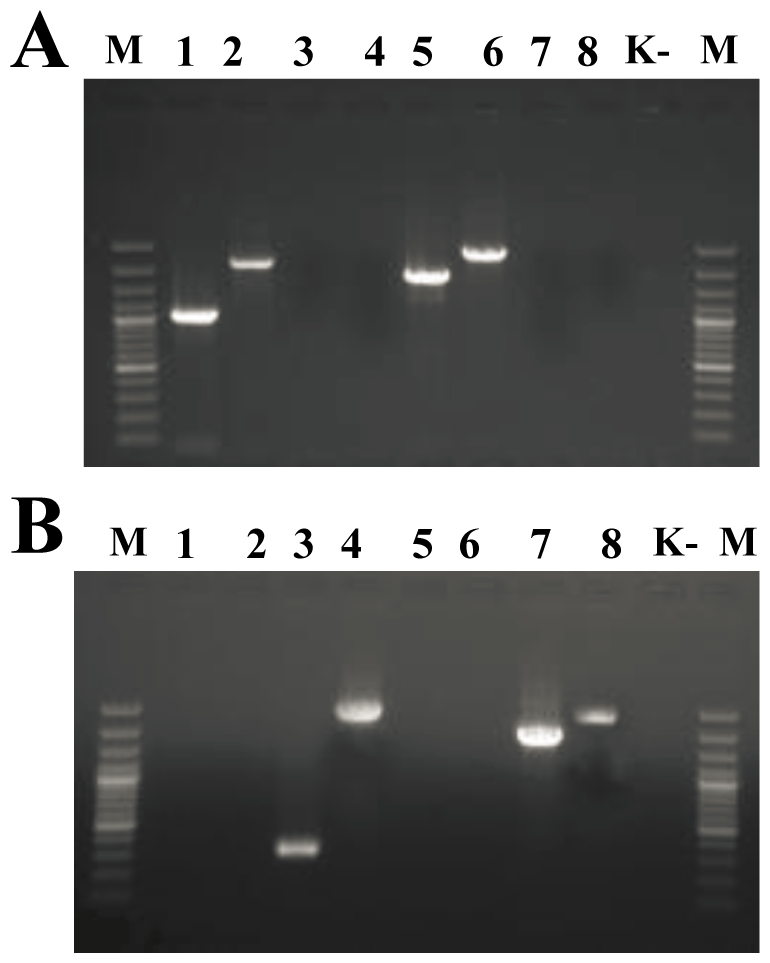
Results of PCR verification of inversions. Electrophoregram of PCR products obtained for MTB strains during the amplification with primer sets 1–8 (Table 3).(A) SP 21 B0/W148 Beijing strain and (B) SP 5 non-B0/W148 Beijing strain. Lanes 1–8 correspond to primer sets 1–8; M is a marker GeneRuler 100 bp Plus DNA Ladder (Fermentas, SM0324); K- is a negative control.

We applied the developed amplification systems followed by sequencing of PCR products to the analysis of 40 MTB strains from the phylogenetic lineages 2 (East-Asian/Beijing) and 4 (Euro-American). Among them, 20 strains belonged to the Beijing family, including 8 Beijing B0/W148-cluster isolates, four strains belonged to Ural, and eight strains belonged to LAM. Primer pairs 1, 2, 5, and 6 (Table 3) amplified PCR fragments exclusively in the Beijing B0/W148 strains yielding 1021, 2215, 1761, and 2527 bp amplicons, respectively (see Figure 4A, lanes 1, 2, 5, 6, as an example). No PCR products were obtained for 32 non- B0/W148 strains (Figure 4B, lanes 1, 2, 5, 6).

On the contrary, the primer pairs 3, 4, 7, and 8 using the same primers in different combinations (Table 3) amplified expected PCR products in non-B0/W148 strains (Figure 4B, lanes 3, 4, 7, and 8), whereas no PCR products were obtained for Beijing B0/W148 strains (Figure 4A, lanes 3, 4, 7, and 8). The differences in length of amplicons produced by primer sets 4, 7, and 8 for groups of non-B0/W148 Beijing and non-Beijing (Ural and LAM) strains (Table 3) is related to the presence of a complete copy of IS*6110* in the analyzed region in non-B0/W148 Beijing strains in contrast to LAM and Ural strains. The specificity of produced PCR products was confirmed by Sanger sequencing in all cases.

These results were additionally verified by using the alternative primer sets selected in the similar way. Primer sequences, expected amplicons' lengths, and electrophoregram of PCR products obtained for Beijing B0/W148 and non-Beijing B0/W148 strains are presented in the Text S2.

Thus, we demonstrated the presence of identical inversions in chromosomal DNA of the studied Beijing B0/W148 strains (n = 8), which appears to be a unique event specific to this clonal cluster.

### The hypothetical reconstruction of recombination events in Beijing B0/W148 progenitor

**Figure 5:**
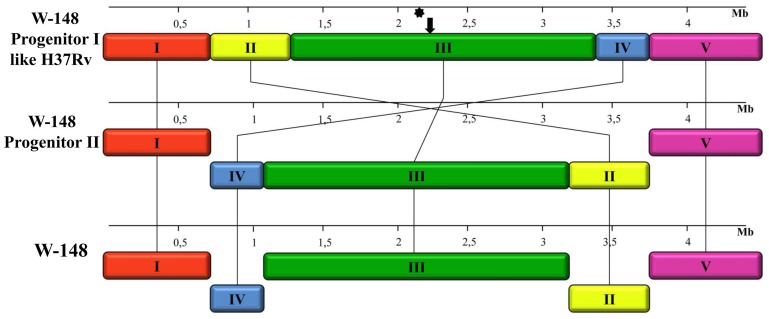
Genome rearrangements' representation for W-148 Progenitor I like H37Rv, W-148 Progenitor II and W-148 genomes. Each local collinear block (LCB) I–V is represented by a different color. Upside-down blocks (LCBs II and IV) represent the location of the reverse strand, which means an inversion has occurred. Asterisk indicates a terminus of a replication site. Terminus of a replication site was calculated based on GraphDNA (GC-skew mode) software [19].

Further we tried to reconstruct the order of rearrangements occurred in a hypothetical W-148 progenitor genome. We suggested that the order and orientation of LCBs in the genome of the W-148 progenitor 1 (P1) was the same as in H37Rv and in other Beijing strains and designed it *in silico* (Figure 5). During the evolution, the first 3 Mb inversion occurred symmetrically across the replication axis and affected LCBs II, III and IV with the formation of progenitor 2 (P2). This recombination event rearranged chromosomal DNA between Rv0609a and Rv3327 genes relative to H37Rv (Figure 2). However, in other Beijing strains, the region between Rv3326 and Rv3327 genes was already disrupted by integration of IS*6110*. Interestingly, we found only parts of IS*6110* in recombination junctions of the inverted region in the genome of W-148. The 812-bp and 543-bp fragments of IS*6110* were detected at the boundaries of the LCBs I&II and LCBs IV&V, respectively. These two parts were inverted and formed together a perfect whole sequence of IS*6110*. We suppose that P1 had two inverted copies of IS*6110*, which were integrated into sites equidistant from the terminus of a replication (*ter*) region.

According to our hypothesis next step of recombination occurred in Progenitor 2 genome and affected LCB III between Rv3020c and disrupted Rv1135c genes. This inversion restored an original orientation of this segment to the initial form like in P1 and H37Rv and led to the formation of W-148. The inversion of this LCB was most probably mediated by two inverted complete copies of IS*6110*, which were found on the borders of this LCB. Remarkably, all Beijing strains in the NCBI database have a complete copy of IS*6110* between LCBs II and III (between Rv3019 and Rv3020c genes), between LCBs III and IV (disrupted the Rv1135c gene), and between LCBs IV and V (between Rv3326 and Rv3327 genes), while they do not have it between LCBs I and II (between Rv0609 and Rv0610c genes).

## Discussion

This study focused on the genomic characterization of the MTB strains of the Beijing B0/W148 cluster, endemic for Russia and representing the epidemiologically successful variant of MTB [2], [5], [6]. Recently, Mokrousov [2] hypothesized that “B0/W148 likely originated in Siberia, and its primary dispersal was driven by a massive population outflow from Siberia to European Russia in the 1960–80s” and “a historically recent, phylogenetically demonstrated successful dissemination of the Beijing B0/W148 strain was triggered by an advent and wide use of the modern anti-TB drugs and was due to its remarkable capacity to acquire drug resistance”. For this reason, we sequenced genomes of four Beijing B0/W148 MTB clinical strains isolated in Russia in 2010–2011.

We used the 454 pyrosequencing technology, which produces the long reads (up to 800 bp). This gave us a good opportunity to see indels, and to identify most of the LSPs (large sequence polymorphisms) present in the studied genomes. Additionally, the genome sequence of W-148 strain represented in GenBank was included in analysis.

Comparing genomes of H37Rv and W-148 strains, we detected two large-scale inversions in the genome of W-148, which were confirmed to be present in all studied strains of Beijing B0/W148-cluster. Notably, the presence of large-scale chromosomal rearrangements within mycobacteria genus was recently shown by *in silico* analysis [20]. The genome of *Mycobacterium smegmatis* mc(2) 155 contains a 56 Kb duplicated region when compared with ATCC 607 progenitor. This duplication is flanked by two copies of an IS*1096* element [21]. Comparative genomics revealed two large tandem chromosomal duplications, DU1 and DU2, in *Mycobacterium bovis* BCG strain. DU1 was found only in BCG Pasteur, while four different forms of DU2 were found in different BCG strains [22]. Two cases of large duplications occurred in the MTB belonged to lineages 2 and 4 have been reported to date [10], [11]. Some of the duplicated regions were flanked by IS*6110* elements supporting a general assertion that the majority of genomic rearrangements are mediated by the mobile genetic elements or repeats [23].

As far as large-scale chromosomal inversions are concerned, a single event was detected among *M. tuberculosis* KZN strains, and there were several such events in *Mycobacterium avium* evolution. Three KZN strains sequenced by Broad Institute showed a large-scale inversion of nearly 2.5 Mb (spanning coordinates ∼1 Mb to ∼3.5 Mb, relative to the origin of replication), although the re-sequencing of one of these strains in another laboratory found no evidence for this event [24]. In *M. avium*, large-scale inversions were found between *M. avium* subspecies *hominissuis* and subspecies *paratuberculosis* [25]. The interspecies comparison of genomes of fish *M. marinum* isolates and *M. tuberculosis* also revealed X-shaped chromosomal inversions derived from the accumulation of rearrangements that were symmetrical across the replication axis [26].

In our study, we discovered the large-scale chromosomal rearrangements characteristic for MTB isolates of the Beijing B0/W148-cluster. The presence of these inversions in all members of Beijing B0/W148 group was confirmed by PCR, sequencing and RFLP hybridization analysis. Additionally, we suggest a two-step scenario of evolution for these strains. In the first step, a large-scale inversion of the 3 Mb segment of the chromosome occurred. This assumption is based on the fact that boundaries of inversion are perfectly equidistant from the site of terminus of replication (i.e., symmetrical across the replication axis). There is a lot of data supporting the chromosome rearrangement around the *ter* region in other bacterial genomes [27], and MTB may have probably implemented a similar mechanism. However, the reason why we have found only half of IS*6110* at the boundaries of inversion is not clear. Remarkably, one part of this disrupted IS*6110* contains a site for *PvuII* while its second part contains the sequence used as a probe in IS*6110*-RFLP typing (between LCBs I and II), which is why only one band is detected in the IS*6110-*RFLP profile. This ∼7.4 Kb band corresponds to two sites for *PvuII* found in unique regions of the W-148 genome (Figure S2). Using the BioNumerics version 5.1 package we compared a collection of IS*6110* RFLP profiles of different Beijing and non-Beijing genotypes. As a result, only members of the Beijing B0/W148-cluster contained the ∼7.4 Kb band demonstrating their unique origine.

**Figure 6:**
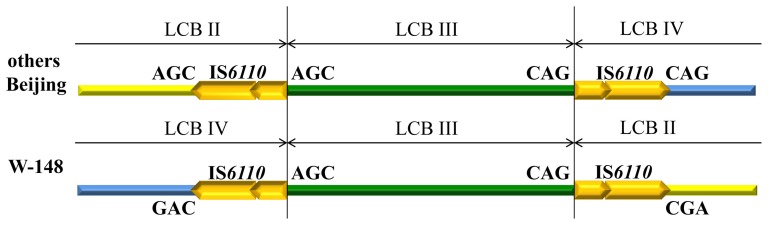
Schematic presentation of duplicated bases flanking the IS*6110* insertion sites between LCBs II&III and III&IV.

The second inversion occurred with LCB III and partly restored an orientation of the large inner segment. As it was noted above, the IS*6110* flanking LCB III is found in all Beijing strains available in GenBank. One of the characteristics of IS*6110* integration is a duplication of the 3–4 base pair region flanking the inserted element at the insertion site [28]. We checked the presence of these duplications in the genomes of B0/W148 and non-B0/W148 Beijing strains. In non-B0/W148 strains, the duplication of AGC proximal to the IS*6110* insertion site between LCBs II and III was found, while the CAG was duplicated between LCBs III and IV (Figure 6). In B0/W148 strains, the sequences of duplicated triplets in the LCB III were in the same orientation, while the sequences of triplets in the LCBs II and IV were inverted and rearranged, which corresponded to the origin of W-148 from W-148 Progenitor 2 (Figure 6). In this case, a homologous recombination between IS*6110* elements appears to be the most appropriate mechanism for the inversion.

Another possible evolutionary scenario suggests that LCBs II and IV have recombined independently of LCB III. According to this hypothesis, LCBs II and IV could recombine simultaneously or stepwise. However, it seems unlikely that blocks II and IV were involved in two independent recombination events simultaneously. Thus, it should have been a sequential recombination process. At first, block II or IV recombined and then the remaining one. Since these blocks are very distantly located from each other, these recombination events most likely were independent. The case where LCBs II and IV have recombined independently of LCB III is also possible but looks improbable.

It has not escaped our notice that the described large rearrangements could have potential consequences for the phenotype as described for other bacteria [29]. Therefore we looked more closely at genes involved into the postulated recombination events. As described in Results section above, the discovered inversions occurred in the proximity of the Rv0609, Rv0610c, Rv1135c, Rv3019, Rv3020c, Rv3326, and Rv3327 genes (Figure 2). However, only in the B0/W148 strains the disrupted part of IS*6110* element is found between Rv0609 and Rv0610c in comparison with other Beijing strains. Both of these genes code for hypothetical proteins, they are located far away from the site of recombination, and it is hard to assume any influence on the phenotype.

To understand the causes of recombination events in Beijing B0/W148 strains, the complete list of unique cluster-specific SNPs (n = 94) was built (Table S1). We have included only those mutations which were found in at least four of the five isolates under consideration. All of these SNPs were mapped to genes coding the proteins of repair, recombination and replication (3R) system in the MTB [30], [31]. No non-synonymous SNPs were found. One synonymous mutation Gly269Gly was found in the RecF protein, which could hardly be associated to large-scale inversions.

To classify the precise genetic sublineage of our sequenced strains, we examined five specific LSPs present in genomes of the East Asian lineage (RD105, RD207, RD181, RD150 and RD142) [32], [33]. According to this analysis, the studied strains belong to the Beijing sublineage 3 (RD105, RD207 and RD181 were deleted), as well as the strains with the large duplications recently reported [10], [11]. These studies reported the large duplication occurred in the strains within sublineages 3, 4 and 5, which spans 350 Kb of the chromosome from the Rv3128c to the Rv3427c genes. Additionally, this duplication was flanked by two complete copies of IS*6110* in the same orientation. After a detailed review of strains studied, we found no evidence of IS*6110* duplication, and the different location of inversions boundaries. Remarkably, according to Weiner *et al.* [11] the strain T67 had downstream boundary at *Rv3326*, and it corresponded to the boundary of the LCB V in the W-148 genome. Interestingly, this region additionally corresponds to the RvD5 in the H37Rv genome and Rv3326, which is a part of IS*6110* flanking it from one side [34], [35]. In almost all Beijing strains, the orientation of IS*6110* in this region is different from the H37Rv.

In summary, we described a chromosomal rearrangement, inversions of large DNA segments in strains of the MTB Beijing B0/W148-cluster. The members of this group possess particular pathobiological features mentioned above, and further studies are necessary to determine the impact of the found inversions on the biological properties of the pathogen. These and previously described inversions and previously reported duplications in the region from 3 Mb to 4 Mb are intriguing and cause an increased interest in these genomes. These rearrangements may possibly reflect evolution of the global chromosomal DNA topology or local DNA-DNA interactions within this region. We hope that our study and studies of other bacteria concerning large-scale rearrangements will shed light on the understanding of the genome evolution of MTB.

## Materials and Methods

### Mycobacterial isolates

A total of 40 MTB strains were obtained from the culture collection of the Research Institute of Phtisiopulmonology (St. Petersburg, Russia) and Moscow Scientific-Practical Center of Treatment of Tuberculosis of Moscow Healthcare (Moscow, Russia). The susceptibility testing was done using a BACTEC™ MGIT™ 960 Culture system (Becton Dickinson, USA) by standard protocol. Standard MTB genotyping methods, including spoligotyping and 24 loci MIRU-VNTR were applied to these strains as previously described [16], [36] (Table S2). Of them 28, 8, and 4 isolates had Beijing, LAM, and Ural spoligotype profiles, respectively. For Beijing isolates with spoligotype SIT1 IS*6110* RFLP analysis was additionally performed [37]. BioNumerics version 5.1 package (Applied Maths, Belgium) was used for band comparison. According to RFLP analysis eight isolates belonged to Beijing B0/W148 cluster. Four of them were selected for a current whole genome re-sequencing project (Table 1).

### Whole genome sequencing and assembly

DNA extraction was performed as previously described [37]. Four B0/W148 strains, SP1, SP7, SP21, and MOS11 were sequenced by using the Roche 454 Life Sciences Genome Sequencer FLX following the manufacturer's instructions (Roche 454 Life Science, USA). Assembly of raw sequencing reads with an average length of 540 bases was performed by the GS de novo assembly software version 2.8 (Roche 454 Life Science, USA). Raw sequencing data for MTB genomes SP1, SP7, Sp21, and MOS11 were deposited in the NCBI Sequence Read Archive (http://www.ncbi.nlm.nih.gov/Traces/sra/) under accession numbers SRX216883, SRX216889, SRX216899, and SRX216918.

The SP21 MTB strain was sequenced using the Ion Torrent PGM (Life Technologies, USA). Two Ion Torrent PGM mate-paired libraries with average size 2 to 3 Kb and 3 to 4 Kb were constructed using Ion mate-paired library preparation guide (Life Technologies, USA). For genome scaffolding, a hybrid assembly strategy was chosen. Initially, GS de novo assembler was used for the 454 and Ion Torrent data assembly, and further Ion Torrent reads were used for genome scaffolding by SSpace [38]

### Genomes analysis

All individual reads generated using the 454 platform were mapped to H37Rv [GenBank: AL123456.2] genome using the 454 GS Reference Mapper (Roche 454 Life Science, USA). Consensus sequences were called, and point mutations identified for sites covered by at least 3 reads, with PHRED scores greater than 30. SNPs calling for whole genomes and contigs from GenBank database representative of the Beijing genotype, 02_1987 (ABLM00000000), 94_M4241A (ABLL00000000), CCDC5079 (CP001641), CCDC5180 (CP001642), CTRI-4 (AIIE00000000.1), HN878 (ADNF00000000.1), R1207 (ADNH00000000.1), Strain_210 (ADAB00000000), T85 (ABOW00000000), W-148 (GL877853.1), and X122 (ADNG00000000.1) was done using MUMmer 3.20 with its nucmer and show-snps functions [39].

Phylogenetic tree was built based on overall SNPs extracted from genomic DNA sequences after excluding SNPs for PE-PPE and PGRS protein families using MEGA4. *M. canettii* was taken as an out-group. A Neighbor-Joining algorithm was used to build a tree. Phylogenetic distance was calculated by using p-distance.

Genomic sequences of four B0/W148 strains under the study and W-148 [GenBank: GL877853.1] were compared to each other and to the H37Rv MTB strain. Genomes were aligned with the open-source MAUVE aligner, version 2.3.1, using the progressive algorithm [40] (http://gel.ahabs.wisc.edu/mauve/).

### PCR verification of inversions

The standard PCR was carried out in 25 µL of reaction mixture. The reaction mixture contained 66 mM Tris–HCl (pH 9.0), 16.6 mM (NH_4_)_2_SO_4_, 2.5 mM MgCl_2_, 250 µM of each dNTP, 1 U of Taq DNA polymerase (Promega, USA), 2.5 mM betain (SIGMA, USA) and 10 pmol of each primer (Table 3, Text S1). One to ten nanograms of genomic DNA were used as a template for PCR. A universal amplification profile included the following steps: the initial heating step was at 94°C for 2 min, followed by 30 cycles of 94°C for 30 sec, 61°C for 15 sec and 72°C for 20 sec and a final step at 72°C for 5 min. The PCR products were then sequenced by conventional Sanger capillary methods on ABI Prism 3730 Genetic Analyzer (Applied Biosystems, USA; Hitachi, Japan) and compared to the H37Rv and W-148 genomes.

### RFLP analysis

RFLP analysis was performed as recommended by van Embden et al. [37] with modifications. Briefly, the whole genomic DNA of SP21 and H37Rv *M. tuberculosis* strains were treated with 15 units of *Mlu*I (Thermo Scientific, USA) in recommend reaction buffer during the night at 37°C. Probes for the Southern analysis were obtained by conventional PCR using Amersham ECL labeling and detection systems (GE Healthcare) with dedicated primers sets (Supplementary Text S2). The obtained profiles on ECL films were scanned and processed with BioNumerics version 5.1 package (Applied Maths, Belgium).

## Supporting Information

Figure S1 **Alignment of genomes of H37Rv, W-148, and SP21 MTB strains represented by Mauve 2.3.1.** Colored outlined blocks surround regions of the genome sequence that aligned to part of another genome (LCBs numbering is the same as in the **Figure 2** of the manuscript). Lines link blocks with homology between genomes. Genomes from top to bottom: H37Rv, W-148, and SP21. Vertical red lines in the SP21 correspond to the boundaries of the scaffolds. Scaffolds 5, 3, and 9 containing sequences of inverted regions are indicated by double-headed arrows. The sequences flanked the sites of inverted regions were found within scaffolds 5, 3, and 9. Scaffold 5 (392,333 bp) includes full sequence of the LCB IV (for LCB numbering and length see Table 2 and Figure 2 in the main text) and parts of the LCB I and III (29 Kb and 16 Kb, respectively). Scaffold 3 (738,393 bp) includes large parts of the LCB III and II (16 Kb and 132 Kb). Scaffold 9 (162,927 bp) includes parts of the LCB II and V (132 Kb and 31 Kb, respectively).
(TIF)

Figure S2 **Schematic view of the IS** ***6110*** **RFLP profiles of the B0/W148 strain generated with Bionumerics program.** M- molecular weights marker (*Pvu*II digested DNA of strain Mt14323). Arrow indicates particular fragment of IS*6110* RFLP corresponding to the inverted region in the Beijing B0/W148 isolates.
(TIF)

Table S1 **B0/W148 cluster specific SNPs.**
(XLSX)

Table S2 **Description of** ***M. tuberculosis*** **clinical isolates involved in this study.**
(XLSX)

Text S1 **RFLP-analysis for confirmation of inversions.**
(DOCX)

Text S2 **Additional primers designed for confirmation of inversions.**
(DOCX)
