# Correction: Unusual Large-Scale Chromosomal Rearrangements in *Mycobacterium tuberculosis* Beijing B0/W148 Cluster Isolates

**DOI:** 10.1371/journal.pone.0091479

**Published:** 2014-02-28

**Authors:** The PLOS ONE Staff

The name of the 14th author is incorrect. The correct name is: Viacheslav Iu Zhuravlev.
