# Supplementary figures and images for: Unusual Large-Scale Chromosomal Rearrangements in Mycobacterium tuberculosis Beijing B0/W148 Cluster Isolates
Source: PLoS One. 2014 Jan 8;9(1):e84971. doi: 10.1371/journal.pone.0084971 (PMC3885621; doi:10.1371/journal.pone.0084971)

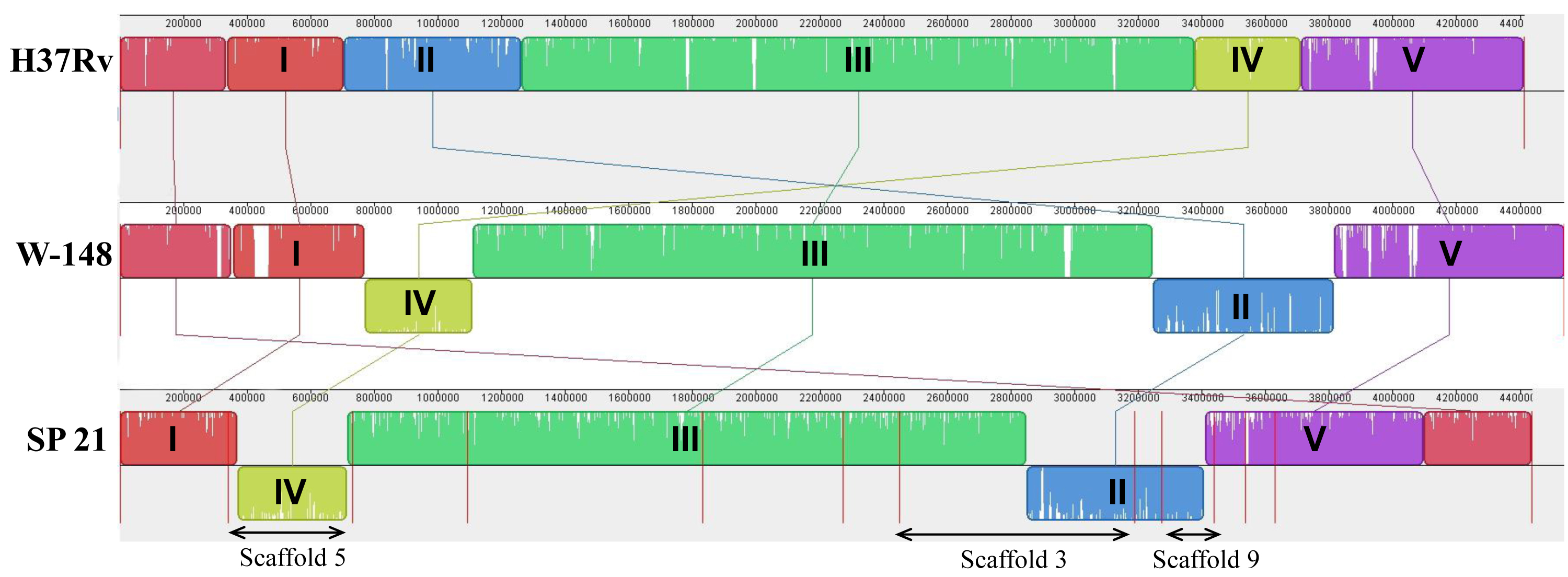

Supplement: Figure S1 — Alignment of genomes of H37Rv, W-148, and SP21 MTB strains represented by Mauve 2.3.1. Colored outlined blocks surround regions of the genome sequence that aligned to part of another genome (LCBs numbering is the same as in the Figure 2 of the manuscript). Lines link blocks with homology between genomes. Genomes from top to bottom: H37Rv, W-148, and SP21. Vertical red lines in the SP21 correspond to the boundaries of the scaffolds. Scaffolds 5, 3, and 9 containing sequences of inverted regions are indicated by double-headed arrows. The sequences flanked the sites of inverted regions were found within scaffolds 5, 3, and 9. Scaffold 5 (392,333 bp) includes full sequence of the LCB IV (for LCB numbering and length see Table 2 and Figure 2 in the main text) and parts of the LCB I and III (29 Kb and 16 Kb, respectively). Scaffold 3 (738,393 bp) includes large parts of the LCB III and II (16 Kb and 132 Kb). Scaffold 9 (162,927 bp) includes parts of the LCB II and V (132 Kb and 31 Kb, respectively). (TIF) [file pone.0084971.s001.tif]
