# Supplementary material for: Unusual Large-Scale Chromosomal Rearrangements in Mycobacterium tuberculosis Beijing B0/W148 Cluster Isolates
Source: PLoS One. 2014 Jan 8;9(1):e84971. doi: 10.1371/journal.pone.0084971 (PMC3885621; doi:10.1371/journal.pone.0084971)
Supplement: Text S1 — RFLP-analysis for confirmation of inversions. (DOCX) [file pone.0084971.s005.docx]

**Text S1.**

RFLP analysis was used for additional confirmation of inversion. RFLP was performed as recommended by van Embden *et al*. [1] with modifications. After *in silico* analysis *Mlu*I restriction enzyme was chosen for DNA digestion, because H37Rv and SP21 genomes have restriction sites, which are not far from recombination junctions (Figure 1). PCR fragments on both boundaries of recombination junctions were used as probes for the Southern hybridization. Fragments were amplified from H37Rv genomic DNA as a template using specific primer pairs (Table 1).

The row hybridization patterns obtained for H37Rv and SP21 strains with each probe are shown in Figure 2. In some lines (B-SP21, C-SP21, F-H37Rv, and F-SP21) we have seen several fragments that we considered as non-specific hybridization products.

**
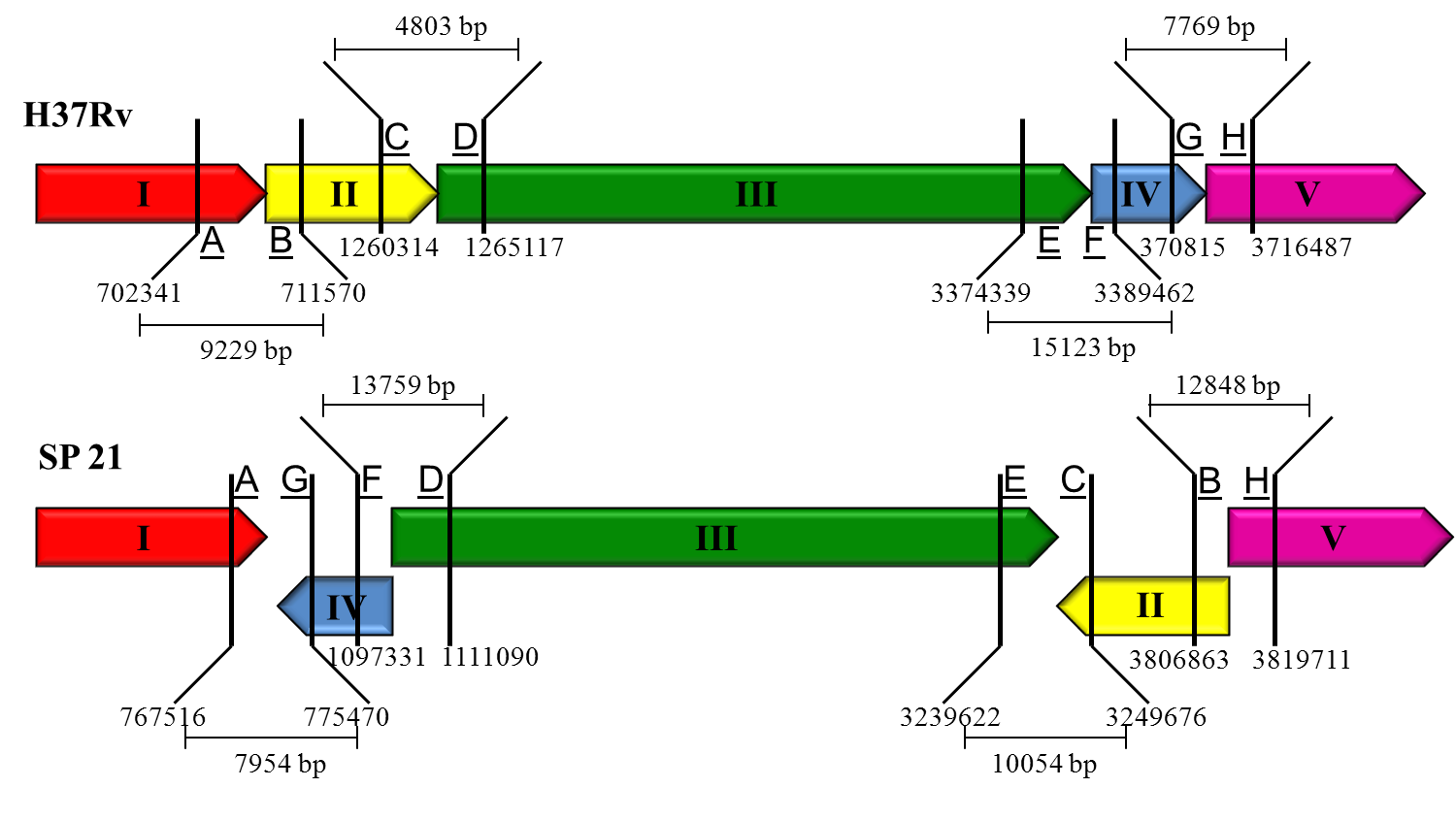
**

Figure 1. **Schematic representation of the RFLP strategy.** Vertical lines indicate the *Mlu*I recognition sites which are close by recombination junctions. Expected lengths of RFLP fragments are indicated between these lines. DNA probes for southern blot hybridization are marked by letters from A to H.

Table 1. Primers for RFLP analysis

| DNA probe | Primers for amplification (5'-3' sequence) | Length |
| --- | --- | --- |
| A | GGCGTTGAACATCAAAGATCC | 412 |
|  | CCAGTGCTCTGCTGATGACC |  |
| B | ACGATGTCCCATAATCGTTGG | 392 |
|  | GAGGCGTCGTGACCGACG |  |
| C | CGAGTGACCGATGAAGAAGG | 343 |
|  | CGCAGGCAACAACAACTTCG |  |
| D | TTGGGAGAGCCGTGATTTGC | 309 |
|  | CGACTACAACCTAGGCAACG |  |
| E | GATCCACCAACAGCGCAACG | 279 |
|  | TGAGCAGGCCGTGCTGTCC |  |
| F | CCTCACCCAAATTGGCTTGC | 262 |
|  | TGGTGATTCGAATTGGAAGG |  |
| G | AACGCCTCCATCTCCACACC | 354 |
|  | GAACGACCACGATGGGGTGC |  |
| H | GGTGAACTTGTCGGGATTGG | 324 |
|  | GGTGACCTGGACACGGTGG |  |


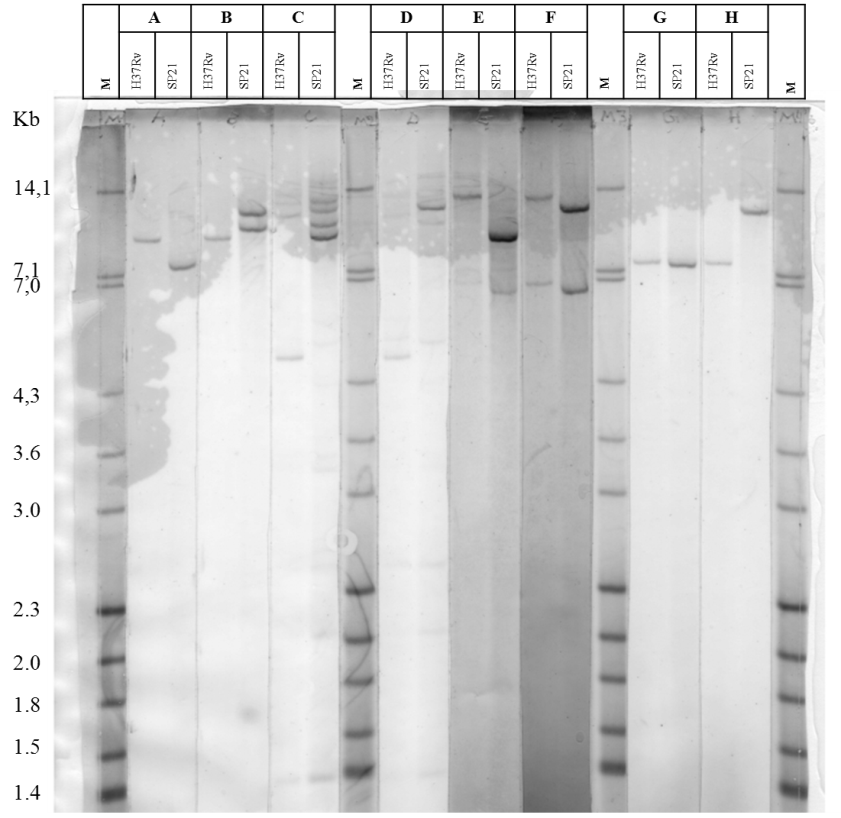


Figure 2. Hybridization of the *Mlu*I-digested DNA of *M. tuberculosis* strains H37Rv and SP21 with different probes (A to H). M, marker strain Mt14323 (Mycobacterial Reference Laboratory, National Public Health Institute (Turku, Finland))

### References

1. van Embden JD, Cave MD, Crawford JT, Dale JW, Eisenach KD, *et al.* (1993) Strain identification of *Mycobacterium tuberculosis* by DNA fingerprinting: recommendations for a standardized methodology. J Clin Microbiol 31: 406-409.
