# Supplementary material for: Unusual Large-Scale Chromosomal Rearrangements in Mycobacterium tuberculosis Beijing B0/W148 Cluster Isolates
Source: PLoS One. 2014 Jan 8;9(1):e84971. doi: 10.1371/journal.pone.0084971 (PMC3885621; doi:10.1371/journal.pone.0084971)

**Text S2.**

On the basis of W-148 genome 8 additional primers’ pairs were designed to verify our data: two pairs (C1&C2, C3&C4) complementary to the regions flanked the ends of the external inversion and two another pairs (C5&C6, C7&C8) complementary to the regions flanked the ends of internal one. Primers’ sequences and amplicons’ lengths expected for B0/W148 and non- B0/W148 strains are presented in the Table S1 and Figure S1. We applied the developed amplification systems on the same strains, which were mentioned in the manuscript. Primers’ pairs C1&C2 and C3&C4 worked with all B0/W148 strains yielded 1708-bp and 2882-bp amplicons, respectively (Table S1, Figure S1 A (lines 1 and 2)). Within non-B0/W148 strains these primers yielded no amplicons, while differently shaped pairs C1&C3 and C2&C4 yielded a PCR product. In case of non-B0/W148 Beijing strains 1063-bp and 3531-bp amplicons were generated (Table S1), whereas for LAM and Ural strains amplicons’ lengths were 1063-bp and 2456-bp, respectively (Table S1, Figure S1 B (lines 3 and 4)).

In a similar manner PCR primers’ pairs C5&C6 and C7&C8 for internal inversion were used. These primers similarly produced bands of 2233-bp and 2220-bp amplicons only in B0/W148 cluster strains (Table S1, Figure S1 A (lines 5 and 6)). Whiles, in non Beijing strains the primer pairs C6&C8 and C5&C7 gave us 1003-bp and 2734-bp amplicons, respectively (Table S1, Figure S1 B (lines 7 and 8)).

**Table S1. Additional primer pairs designed for confirmation of inversion**

| **Primers’ set No** | **Primers’ name** | **5’-3’ - sequence** | **Product’s length** | | |
| --- | --- | --- | --- | --- | --- |
|  |  |  | **B0/W148 Beijing** | **others Beijing** | **non-Beijing** |
| External inversion (links between LCB I, II, IV, V) | | | | | |
| 1 | C1&C2 | CTTGACTCCCAGCGTGATCC | 1708 | no reaction | no reaction |
|  |  | GCACACTCTCGTCGTATTGC |  |  |  |
| 2 | C3&C4 | GAGGCGTCGTGACCGACG | 2882 | no reaction | no reaction |
|  |  | GGCATCACCGGAATTGAAGC |  |  |  |
| 3 | C1&C3 | CTTGACTCCCAGCGTGATCC | no reaction | 1063 | 1063 |
|  |  | GAGGCGTCGTGACCGACG |  |  |  |
| 4 | C2&C4 | GCACACTCTCGTCGTATTGC | no reaction | 3531 | 2456 |
|  |  | GGCATCACCGGAATTGAAGC |  |  |  |
| Internal inversion (links between LCB II, III, IV) | | | | | |
| 5 | C5&C6 | GATCCACCAACAGCGCAACG | 2233 | no reaction | no reaction |
|  |  | CGAGTGACCGATGAAGAAGG |  |  |  |
| 6 | C7&C8 | TGGTGATTCGAATTGGAAGG | 2220 | no reaction | no reaction |
|  |  | CGACTACAACCTAGGCAACG |  |  |  |
| 7 | C6&C8 | CGAGTGACCGATGAAGAAGG | no reaction | 2361 | 1003 |
|  |  | CGACTACAACCTAGGCAACG |  |  |  |
| 8 | C5&C7 | GATCCACCAACAGCGCAACG | no reaction | 2091 | 734 |
|  |  | TGGTGATTCGAATTGGAAGG |  |  |  |

**Figure S1. PCR verification of inversions.** (A) Amplification products of B0/W148 Beijing strain. (B) Amplification products of non-Beijing strain. Lanes 1 - 8 are correspond to primers’ sets 1 – 8, M is a marker GeneRuler 100 bp Plus DNA Ladder (Fermentas, SM0324). K- is a negative control.


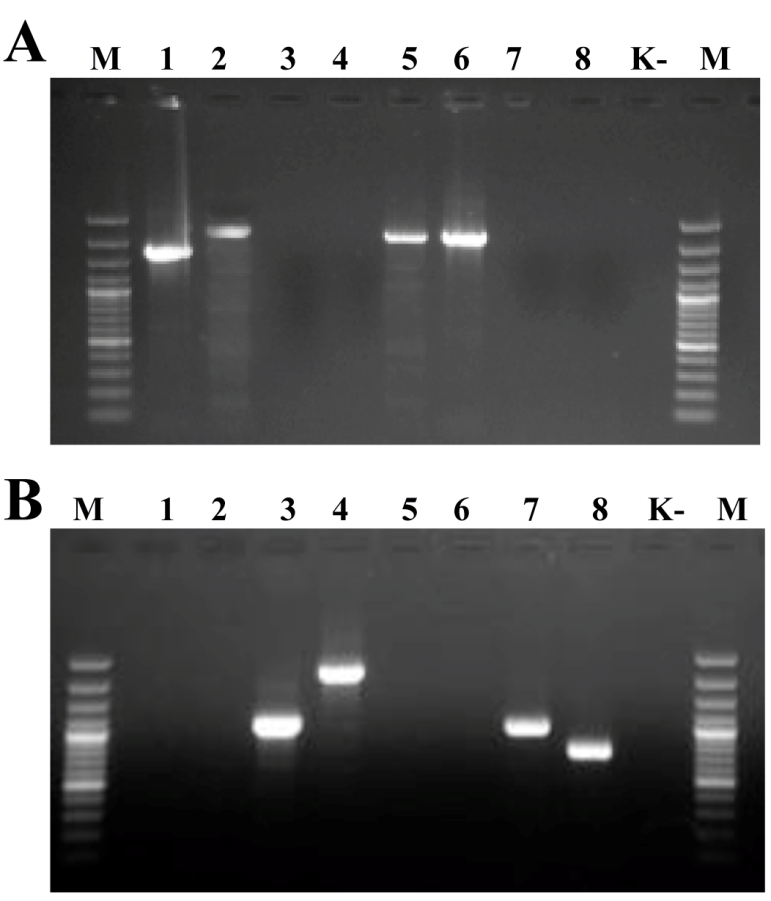

Supplement: Text S2 — Additional primers designed for confirmation of inversions. (DOCX) [file pone.0084971.s006.docx]
